# Gas Plasma-Augmented Wound Healing in Animal Models and Veterinary Medicine

**DOI:** 10.3390/molecules26185682

**Published:** 2021-09-19

**Authors:** Sander Bekeschus, Axel Kramer, Anke Schmidt

**Affiliations:** 1 ZIK *Plasmatis*, Leibniz Institute for Plasma Science and Technology (INP), Felix-Hausdorff-Str. 2, 17489 Greifswald, Germany; anke.schmidt@inp-greifswald.de; 2 Institute for Hygiene and Environmental Medicine, Greifswald University Medical Center, Sauerbruchstr., 17475 Greifswald, Germany; axel.kramer@med.uni-greifswald.de

**Keywords:** chronic wounds, cold physical plasma, infected wounds, plasma discharge, plasma medicine, reactive oxygen species, RNS, ROS

## Abstract

The loss of skin integrity is inevitable in life. Wound healing is a necessary sequence of events to reconstitute the body’s integrity against potentially harmful environmental agents and restore homeostasis. Attempts to improve cutaneous wound healing are therefore as old as humanity itself. Furthermore, nowadays, targeting defective wound healing is of utmost importance in an aging society with underlying diseases such as diabetes and vascular insufficiencies being on the rise. Because chronic wounds’ etiology and specific traits differ, there is widespread polypragmasia in targeting non-healing conditions. Reactive oxygen and nitrogen species (ROS/RNS) are an overarching theme accompanying wound healing and its biological stages. ROS are signaling agents generated by phagocytes to inactivate pathogens. Although ROS/RNS’s central role in the biology of wound healing has long been appreciated, it was only until the recent decade that these agents were explicitly used to target defective wound healing using gas plasma technology. Gas plasma is a physical state of matter and is a partially ionized gas operated at body temperature which generates a plethora of ROS/RNS simultaneously in a spatiotemporally controlled manner. Animal models of wound healing have been vital in driving the development of these wound healing-promoting technologies, and this review summarizes the current knowledge and identifies open ends derived from in vivo wound models under gas plasma therapy. While gas plasma-assisted wound healing in humans has become well established in Europe, veterinary medicine is an emerging field with great potential to improve the lives of suffering animals.

## 1. Introduction

The skin protects the body from environmental stressors, infectious agents, and loss of homeostasis. Being the body’s largest organ in vertebrates, the skin has multifunctional roles due to its complex structure and multiple compartments such as a layer of dead keratinocytes (corneocytes) in the *stratum corneum* and the *stratum lucidum*, *stratum granulosum*, *stratum spinosum*, and *stratum basale* in the epidermis. Below the epidermis, the dermis is host to a rich meshwork of vasculature, immune cells, hair follicles, and glands [1].

**Figure 1:**
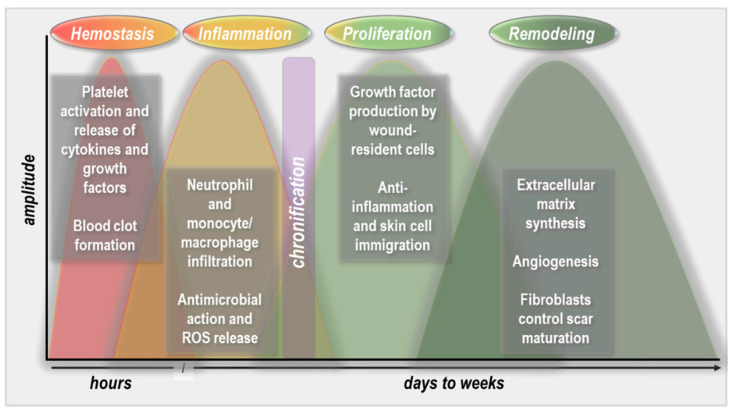
Wound healing stages. During hemostasis, platelets become activated to contribute to blood clot formation and release cytokines and growth factors. This is followed by the inflammation phase, which is characterized by extensive immigration of phagocytes, predominantly neutrophils and macrophages, to promote inflammation and pathogen clearance. During the subsequent proliferation phase, profound anti-inflammatory and growth-promoting processes are induced that allow the immigration of fibroblasts and keratinocytes into the wound bed, guided by growth factor production and gradients. Once the wound is closed, the final step is the remodeling of the skin architecture, which is mainly driven by fibroblasts and extracellular matrix synthesis and accompanied by angiogenesis to restore near-original tissue strength.

Skin trauma is inevitable in life. The skin has extensive renewing capacities in both trauma and constitutive conditions. Constitutively, the epidermis renews every 25 to 70 days in an age-dependent fashion. In non-extensive trauma, wound healing usually occurs within a week, with further cellular proliferation and maturation along with intracutaneous matrix remodeling going on for several days to weeks after that. The wound healing phases include hemostasis, inflammation, proliferation and maturation, and matrix remodeling (Figure 1). The phases have defined yet partially overlapping sequences that have been recently reviewed [2]. Extensive trauma, infection, and underlying disease can extend the time to heal significantly. If healing is not achieved within four weeks despite standard care, the wounds are defined as being chronic, and can persist for several months to years. Such insufficient cutaneous wound healing is a burden to patients and health care systems worldwide. Despite a wealth of non-invasive and invasive options to support healing, a considerable number of wounds are refractory to therapy. An aging society and increased prevalence of widespread diseases like diabetes and vascular insufficiencies add to this issue. Antimicrobial resistance (AMR) is also rising globally [3], making efficient wound therapy a critical issue to address.

Wound healing has a redox dimension [4]. Reactive oxygen species (ROS), which include reactive nitrogen species (RNS), as these mostly contain oxygen, contribute to all stages of healing as signaling molecules and are therefore part of signal transduction processes. For details on primary and secondary ROS/RNS relevant in plasma medicine, the reader is referred to a recent review [5]. ROS/RNS are moreover integral components of the antimicrobial defense elicited by phagocytes [6]. Hence, it appears logical to use therapeutic ROS/RNS supplied exogenously to the wound microenvironment to aid in healing by promoting signaling and anti-infective action. About a decade ago, gas plasma technology began to be systemically investigated globally as a novel tool to target defective wound healing based on clinically relevant findings [7,8,9]. Gas plasma is a partially ionized gas generating a plethora of ROS/RNS simultaneously and in a spatiotemporally-controlled manner [10]. The two main types of devices are gas plasma jets [11] and dielectric barrier discharges (DBDs) [12], with the kINPen MED [13,14] and the SteriPlas [15] being well-described and clinically certified examples of each category. Today, based on clinical evidence, several gas plasma devices have been marketed in Europe as accredited medical products class IIa for dermatological applications with a particular focus on wound healing. Animal models and animal patients have been vital in understanding this technology’s efficacy, safety, and suitability in wound healing promotion and identifying ways to improve clinical response. This review aims at summarizing the current knowledge on animal models in investigating gas plasma-assisted wound healing.

## 2. Wound Healing in Animals and Experimental Models

An array of experimental models is available in veterinary science for studying wound healing. These can relate to mainly regenerative models of healing in species such as starfish, zebrafish, frogs, and salamander, as well as models more closely resembling the human wound healing stages, including rodents, ruminants, and pigs [16]. Details on different wound healing models and species in veterinary science and experimental medicine have been thoroughly reviewed recently [17,18,19,20].

### 2.1. Rodent Models

Rodent models, especially mice and rats, are by far the most used models in wound healing research [16]. Both species are easy to house and breed, and genetic engineering tools are well-established for generating constitutive or conditional knock-out and knock-in variants that allow the molecular mechanisms of wound healing to be deciphered. Moreover, small rodent models benefit from fast birth-to-adulthood times and modest housing costs, facilitating the use of larger groups within experiments to obtain robust statistical analysis. For instance, SCID (severe combined immunodeficient) mice are void of T-cells, and these mice heal wounds as efficiently as non-SCID mice do, suggesting a minor role of T-cells in wound healing [21]. Due to rodent models’ small size, a cost-effective manipulation of metabolic or immunological traits by pharmacological or immunotherapeutic interventions is also possible. As an example, the minor relevance of T-cells in wound healing has also been shown by depleting T-cells experimentally using monoclonal antibodies [22].

Although chronic wound healing models closely resembling human pathology are currently not available, there are several options to induce wounding in general to generate models of acute healing, impaired healing, and semi-chronic wounds, as has been summarized before [23]. Briefly, acute models can be generated using punch biopsies, wound chambers, burns, cold, laser, and pressure ulcers. Impaired healing can be achieved by pharmacological, genetic, physical, or microbial means. Examples are the usage of ionizing radiation, malnutrition, glucocorticoid-mediated immunosuppression, mitomycin C and 5-fluorouracil-mediated dermonecrosis, zinc and vitamin deficiencies, ischemia, wound contamination with Pseudomonas and Staphylococcus strains, and models of diabetes and obesity. Additionally, aging is a parameter suitable to investigate impaired healing [24]. With the emergence of two-photon microscopy, real-time in vivo imaging has much accelerated the knowledge on cellular dynamics in wounds. For instance, Lämmermann and colleagues used a reporter mouse model with fluorescently-tagged neutrophils and macrophages to observe their infiltration and swarming phenome following small-scale laser-induced wounding [25]. For larger-scale wounds, the rabbit ear model has been frequently employed [26,27].

### 2.2. Non-Rodent Models

Tissue repair and regeneration in non-rodent models is a field of intense investigation. The perhaps best-investigated vertebrate model is zebrafish in both larval and adult stages. Zebrafish genetics is well-understood, and many genetic variants and reporter models have been generated [28]. Zebrafish larvae with genetically encoded redox sensors and fluorescent reporters for immune cells have helped researchers to understand the first events during wounding [29]. Immediately after wounding, NADPH (nicotinamide adenine dinucleotide phosphate)-oxidase (NOX)-generated superoxide dismutates to hydrogen peroxide (H_2_O_2_), which serves as a gradient for infiltrating leukocytes [30]. If the ROS/RNS production is inhibited by pharmacological means, leukocyte infiltration into the wound does not occur. These data lead to the hypothesis that gas plasma-released ROS/RNS affects the wound microenvironment’s cellular composition, independent of infection. Adult zebrafish are also an excellent model of wound healing, as demonstrated previously [31]. Apart from fish, large animal models are also frequently employed in wound healing research [32]. In particular, pigs and mini pigs are favorable for dermatological research, as their skin architecture closely resembles that found in humans [33]. Porcine ears are a particularly well-investigated ex vivo model system and are also employed in gas plasma medical research to study the consequences of exogenous agents such as ROS/RNS on the skin [34]. In general, non-rodent in vivo models often require extensive and non-standard infrastructure at most research institutions, such that the majority of gas plasma studies have been performed in rodents.

## 3. Gas Plasma Treatment of Skin and Wounds in Animal and Veterinary Models

Much has been learned on the efficacy, mechanisms, and safety of gas plasma treatment of wounds in rodent models, as outlined in the following sections. In this review, only studies on mammals that have received gas plasma treatment of the skin or skin wounds are included, since other model organisms, such as zebrafish, have only started to be investigated in the gas plasma medicine field [35].

### 3.1. Murine Models of Gas Plasma in Wound Healing

Gas plasma-assisted wound healing has been investigated using various devices and rodent models, mainly mice, rats, and rabbits. Most studies were conducted using mice (Table 1) which received a punch or incision biopsies to generate wounds; gas plasma jets were then used for gas plasma-assisted wound healing. In a study investigating a full-thickness model of ear wounds in hairless but immunocompetent SKH-1 mice, treatment with the atmospheric pressure argon (Ar) plasma jet kINPen accelerated healing and angiogenesis [36], as was also found for other argon plasma jets [37,38]. The healing observed with the kINPen was corroborated by improved tissue oxygenation and microcirculation in superficial and deep layers of the wound [39]. Mechanistically, these findings correlated with increased expression and activation of Nrf2 (nuclear factor erythroid 2–related factor 2) and modulation of inflammation and granulation [40]. The healing responses were guided by changes in focal adhesion complexes and junction protein expression, along with epithelial to mesenchymal and fibroblast to myofibroblast transitions (EMT, FMT) [41]. Notably, a one-year follow-up study in those mice, which translated to a 60 year equivalent time span for humans, did not find any side effects or risks associated with carcinogenesis or defective healing [42]. The device is also commercially available as a class II medical product in the EU, the kINPen MED [13]. Another study using a similar mouse model but a different gas plasma jet suggested that feed gas conditions other than pure argon might be more beneficial in mediating wound healing [43]. The wound healing-promoting effect of an argon plasma jet was enhanced if the number of gas plasma treatment sessions was adapted [44], as it was shown that long gas plasma exposure times, in general, can lead to wound necrosis [45]. Leveled gas plasma exposure, by contrast, supports re-epithelization [46] and changes in collagen and vimentin [47] and αSMA (alpha-smooth muscle actin) deposition [48]. Especially for nitrogen (N_2_) plasma jets, positive effects on wound contraction were observed [49], which was accompanied by elevated RNS deposition [50] and laminin production, along with decreased MMP (matrix metalloproteinase)-3 expression [51]. Additionally, helium (He) plasma jets accelerate wound healing [52,53]. Independent of the feed gas, a decreased microbial burden of the natural wound flora [54] and experimentally infected wounds were observed [55]. One study found argon and helium plasma jet-mediated healing to be superior to DBD treatment [56]. In general, jets seem to be preferred to DBD devices in experimental wound healing mouse models, presumably because jets can penetrate small cavities and have pen-like operated guidance while DBDs ‘hide’ the treated surface, making direct proof of uniform plasma discharge treatment difficult. An alternative is gas-driven DBDs with a ROS/RNS-rich afterglow. For instance, an experimental argon plasma torch, the prototype of the commercially available SteriPlas device [15], successfully improved wound healing and modulated inflammation [57] while simultaneously spurring angiogenesis and growth [58].

Apart from the direct gas plasma treatment of incisional wounds of healthy mice, other mouse models have been reported in gas plasma wound healing medicine. For instance, gas plasma-supported wound healing was observed for a helium plasma jet in diabetic mice [65], which was found to be enhanced when adding low amounts of oxygen (O_2_) [69]. For an argon plasma jet, beneficial healing was also observed for burn wounds and infected burn wounds [68]. It is critical to design the gas plasma source with care to keep it at body temperature, as gas plasma jets exceeding those temperatures readily induce burns themselves at longer treatment times and inappropriate gas fluxes [64]. Clinically, burns are often therapeutically addressed using skin grafts. One study investigated the effect of gas plasma treatment on supporting skin engraftment and found elevated angiogenesis and growth factors at the grafted sites [61]. Such a procedure often leads to intense scar formation, and another study focused on gas plasma treatment of scars, finding that scar thickness and vascularization were reduced due to the treatment [66]. Traditional wound treatments involve lavaging with liquids and ointments with creams, oils, or hydrogels. Accordingly, several studies have investigated the effect of gas plasma-treated liquids, oils, and hydrogels compared to non-treated counterparts on wound closure. For hydrogels formed using gas plasma-treated liquids, improved wound healing was observed in random-pattern skin-flap full-thickness wounds [67]. In other models, gas plasma-treated liquids were used as lavage. While one study found beneficial effects of direct gas plasma jet treatment and jet-treated liquids on wound healing [59], another report did not observe such results [60]. Both studies were done in Balb/c mice, with the former using helium and the latter an argon plasma jet. From the findings above, it can be concluded that the lack of findings must be owed to device- or wound model-specific traits, as gas plasma-supported wound healing has been observed for most feed gas conditions so far. In approaches of DBD-treated water used for wound healing, positive results on wound healing and antimicrobial efficacy were achieved [70] that were related to changes in the inflammatory profiles of the wounds [71]. One further study used gas plasma jet-treated oil emulsion to identify improved wound closure upon application [72]. A complimentary report applied oil to the wound first, followed by Ar/air or Ar/O_2_ plasma jet treatment, also reporting positive results [73]. In contrast, the combination of gas plasma jet wound treatment and medical honey did not lead to beneficial healing effects [62,63].

From the findings mentioned above, it can be concluded that gas plasma treatment supports wound healing independent of its antimicrobial efficacy. Even more, gas plasma treatment times or energy deposition need to be adjusted carefully to remain within the therapeutic window of wound healing promotion. Mechanistically, it appears that gas plasma exposure accelerates the sequence and intensity of regular wound healing phases, unambiguously demonstrating that wound healing is subject to redox control that can be targeted by gas plasma-derived ROS/RNS. Improved wound healing was found in all mouse strains investigated and was largely independent of the specific type of device used. In general, it appeared that direct gas plasma treatment of wounds is more promising than the application of gas plasma-treated vehicles such as liquids and oils, as these will quickly deteriorate the short-lived ROS/RNS that make gas plasmas so unique.

### 3.2. Rat and Rabbit Models of Gas Plasma in Wound Healing

Rat and rabbit wound healing models are favorable because their skin is thicker, mimicking human wound healing to a greater extent compared to mice. Furthermore, their larger body surface allows the generation of larger wound areas, which is less conceivable and ethically acceptable in mice. We identified six studies using helium plasma jets and four using argon plasma jets which reported that gas plasma-mediated wound healing was advantageous (Table 2). All argon plasma jet studies were done in Sprague Dawley rats and found increased re-epithelization and less fibrosis in incisional wounds [89], faster wound closure in healthy rats as well as Type I and Type II diabetic animals accompanied by accelerated re-epithelization and fewer neutrophils [90], and decelerated injury progression regarding necrosis and neutrophil influx in burn wounds [91]. Additionally, elevated levels of superoxide dismutates (SOD), catalase (CAT), and glutathione peroxidases (GPx) were found, linking the responses identified to redox control. Interestingly, the fourth study found a helium/argon mixture to be superior to helium alone in terms of improved granulation tissue formation and wound healing, but only for short treatment times [92]. Overall similar results were obtained for helium plasma jets. The treatment accelerated wound healing in both non-diabetic [93] and diabetic animals [94], improved angiogenesis and neovascularization [95], amended wound contraction and epithelization [96], and decreased collagen deposition and scar formation [97].

Five studies using DBSs were done to investigate wound healing in Wistar rats. Improved wound healing and attenuated long-term inflammation were observed [100], which was confirmed in diabetic animals, accompanied by increased epidermal thickness, re-epithelization, collagen deposition, angiogenesis, proliferation, TGF-β (tumor growth factor-beta) release, and an increased number of fibroblasts [101]. Apart from non-infected wounds, augmented healing was also observed in gas plasma-treated infected wounds [100]. Mechanistically, one study identified an increase of free thiols and neutrophil activity, as observed by increased cellular influx and myeloperoxidase (MPO) levels [98]. Gas plasma was also used as an enabling technology, functionalizing polypropylene particles to increasingly absorb betaine hydrochloride, which improved wound healing in diabetic rats [102]. In New Zealand white rabbits, DBD-treated wounds reduced the microbial burden of methicillin-resistant *Staphylococcus aureus* (MRSA) and accelerated re-epithelization and healing [103]. In another study using the same rodent species, DBD-treated wounds led to markedly elevated leukocyte infiltrates within short times following plasma exposure, suggesting plasma-derived ROS/RNS serve as chemoattractants for immune cells that may help to facilitate faster healing responses [104]. One study in diabetic rabbits reported gas plasma jet treatment of the skin to alter the tender structure in joints of the animals [105]. The results are questionable, as it is unclear how ROS/RNS or any other gas plasma agents should be able to directly penetrate such long distances through skin and muscles to reach such targets. More conclusive studies on the effects of gas plasma on the skin have been performed in mice, as described in the following.

### 3.3. Animal Models of Gas Plasma Treatment of Intact Skin

Besides wound healing, an array of studies have investigated the effects of gas plasma on the skin in live animals, predominantly using mice (Table 1). Treating healthy murine skin, Arndt and colleagues used 129Sv/Ev mice and an argon plasma torch as a prototype of the commercially available SteriPlas device [15]. They did not observe any changes in keratinocytes’ proliferation, migration, or apoptosis, concluding the device to be safe [74]. In addition, they found β-defensins, a class of host defense peptides characterized by their antimicrobial activity and capacity for immune stimulation, to be increased. Experiments using a different type of argon-driven DBD found, in addition, an enhanced expression of cytokines and chemokines, such as TGF-β, VEGF (vascular endothelial growth factor), GM-CSF (granulocyte/macrophage colony-stimulating factor), and EGF (epidermal growth factor), along with enhanced epidermal thickness [82]. A lack of side effects, inflammation, or collagen production was also found for another DBD that moreover led to increased NO (nitric oxide) deposition into the tissues [76]. With an air DBD, increased uptake of the anesthetic lidocaine in the skin was observed following gas plasma exposure [84]. For gas plasma jets, kINPen treatment increased the uptake of the model drug curcumin concomitant with altered gene and protein expression of junctional proteins and led to elevated tissue oxygenation and microcirculation [86]. In addition, this first report on a lipidomics analysis of murine gas plasma-treated skin revealed changes in lipid composition in the upper layers of the *stratum corneum*, which was corroborated in a different study finding decreased E-cadherin expression and increased EGF absorption [81]. These studies and the following study were performed in nude, immunocompetent mice. Here, kINPen treatment did not induce dermal degeneration and increased the levels of Nrf2 and catalase in the skin [83]. Questionable results were reported in Balb/c mice with induced diabetes, with study results stating that local skin treatment changed the levels of malondialdehyde (MDA) and the overall advanced oxidation protein products (AOPP) in the blood circulation, even at longer times following treatment [77]. We did not find these results for an argon plasma jet [83]. From these studies, it can be concluded that gas plasma treatment of murine healthy skin was well-tolerated and changed the composition of proteins and lipids, ultimately leading to enhanced drug uptake. Such findings were reported more than a decade ago in human and pig skin [106,107], constituting a promising novel application for gas plasma in dermatology.

Another field of interest is the gas plasma treatment of pathological skin conditions, such as dermatitis and psoriasis. Psoriatic lesions and epidermal thickness were found to be decreased using a He/O_2_ plasma jet [75], which was also confirmed for argon and N_2_ plasma jet conditions [88]. In addition, another study found decreased leukocyte infiltrates and abrogated expression psoriasis-associated inflammatory mediators, such as IL (interleukin)-17 and IL-22 [79]. These are promising results, since psoriatic patients often experience therapy-resistant lesions, making gas plasma a valuable option in those patients. Although the first clinical case reports were only partially encouraging in this regard [108,109], several parameters may be modulated, e.g., gas plasma source, feed gas, treatment time, treatment frequency, and combination therapy, to support the treatment of this disease. A skin condition also investigated for gas plasma therapy effects is atopic dermatitis. In a DBD model, humidified argon was found to best decrease disease severity compared to N_2_ or O_2_ plasma in Balb/c mice [78]. A more detailed study investigated disease immuno-infiltrates and identified decreased numbers of mast cells and eosinophils along with lower epidermal thickness, all of which are associated with disease severity [85]. A report on gas plasma-treated water also suggested this agent could actively reduce the harshness of the disease [104]. Since mainly long-lived ROS/RNS such as H_2_O_2_ and NO_3_^−^ are present in such liquids [5], it is likely that the results might be recapitulated using chemically enriched liquids without the gas plasma process. In terms of hypersensitive skin, allergy is another condition causing patient discomfort. To investigate whether gas plasma treatment causes skin allergies, the kINPen was used to treat the skin of CBA mice. Comparison against allergy-inducing agents as positive controls showed no allergic response to the gas plasma exposure [80]. These results showed gas plasma treatment to be well-tolerated and safe and suggested possible indications of this technology apart from wound healing in dermatology.

### 3.4. Larger Animal Models and Veterinary Patients of Wound Treatment

Several studies have been conducted in larger animal models to investigate the effects of gas plasma exposure on wound healing (Table 3). In two studies using Bergamasca sheep, a novel helium plasma torch designed explicitly for veterinary purposes was studied [110]. The advantage of the source is its large treatment surface area, reducing the long exposure times required for larger wounds. In the first study, improved healing of experimental wounds was observed [110] along with a lower microbial burden. Subsequent wound tissue analysis revealed higher cell proliferation and VEGF presence in gas plasma-treated wounds than controls during early wound healing phases. The results were re-iterated in a follow-up study that included an additional group receiving mesenchymal stem cells (MSCs) [111]. Interestingly, the combination of both approaches led to the best wound healing results. The results may be interesting for small sheep farms that rely on wool production throughout animals’ lifetimes.

Two studies investigated the effects of gas plasma treatment on porcine skin and skin wounds in vivo. In the first study, a floating-electrode DBD was used at different energy intensities and treatment times. Extended exposure times and energies led to tissue necrosis and thermal damage, similar to burns. By contrast, modest treatment modalities did not harm the tissue or wounds being treated. There was no conclusion on overall healing responses. Interestingly, the gas plasma treatment led to rapid hemostasis in pig wounds, a finding that was recently confirmed quantitatively in mice [119] and mechanistically in human donor blood [120,121] using the kINPen argon plasma jet. In another study using this jet, superficial and deep skin wounds in live pigs were exposed to gas plasma [9]. It was found that both untreated and gas plasma-treated wounds healed equally well and that there were fewer inflammatory cell infiltrates in fully healed tissues of the gas plasma group.

Several reports have been published on animal patients in veterinary medicine, mainly cats and dogs. In a case report using the kINPen VET, the only medical product-accredited and commercially available gas plasma device dedicated to veterinary medicine, a dog suffering from cutaneous *Alternaria* spp. infection experienced complete clinical remission after several gas plasma therapy sessions. This led to the cessation of immunosuppressive drugs that the dog had to take during the long time of persistent infection and improved its life quality [113]. Two more extensive studies in 40 and 85 dogs, respectively, did not confirm a dedicated antimicrobial effect of gas plasma in canine bite wounds, however [114,115]. Comparators were saline and polyhexanide, with the latter showing the best antimicrobial results. There was no conclusion on the overall wound healing performance in this first prospective blinded randomized clinical trial in veterinary medicine reported so far. However, the wounds were not characterized as being chronic by definition. In a case report series of eight cats or dogs suffering from therapy-resistant wounds for months to years, repeated treatment with the kINPen led to complete wound remission in seven pet patients and partial remission in all eight patients [116,117]. Compared to initial wound sizes and conditions, the results were remarkable and essential as a proof-of-concept that gas plasma can heal chronic wounds. A second case-cohort of pets suffering from insufficient wound healing re-iterated these findings. All 12 pet patients experienced complete wound healing in the course of gas plasma therapy using the kINPen [116]. These results are promising for using gas plasma in veterinary medicine, especially when targeting problematic wounds resistant to standard-of-care.

## 4. Opportunities of Gas Plasma in Wound Healing Science and Veterinary Medicine

The in vivo studies on gas plasma-aided wound healing in rodents and non-rodents are highly promising for clinical applications. Two main routes can be identified for future research in this field. The first is the further improvement of wound-healing responses using gas plasma devices in human patients. The second is the widespread use of gas plasma technology in veterinary medicine, which so far is underacknowledged.

Despite the clinical success of medical gas plasma devices in wound healing [122,123,124,125,126,127,128,129,130,131,132,133,134,135,136,137,138,139], which goes beyond the reported clinical trials as evidenced by hundreds of devices that were successfully operated in daily practice in many dermatological centers across Europe, several questions remain not fully addressed. Some examples are the identification of ideal exposure times, exposure frequencies onset and offset of therapy, combination with existing wound healing methods, and type of devices to be used that need to be stratified against patient and wound characteristics such as age, underlying disease, medication, wound location, wound size, wound history, infection status, and immunological fitness. Certainly, utilizing small animal models will help to understand such causal relations better. However, strategic designs are necessary to help implement these clinical questions to a greater extent, especially using gas plasma setups already available in the clinics. Additionally, model complexity should be increased to account for the clinical situation, e.g., using a diabetic model of full-thickness wounds contaminated with multi-species biofilms.

It goes without saying that veterinary medicine offers tremendous potential for gas plasma-supported wound healing. Defective wound healing is a prominent issue across several disciplines of this field [140,141], such as farming, pets, and animals used in sports, e.g., racing horses and camels. The economic impact of non-healing wounds is not to be underestimated, as these often require isolation of the animals, repeated visits by veterinarians, and in the case of sports, a lack of participation in competitive events. Proper wound healing is an intense field of research in veterinary sciences [142,143], but—apart from the few cited reports from Germany—published reports on systemic studies or case reports are scarce. The question remains whether this is due to the lack of awareness of the promising traits of gas plasma technology or the lack of appropriate and commercially available large-area devices to target extensive wounds. Another aspect possibly complicating the translation of preclinical and human clinical results to veterinary medicine is potential differences in the wound healing physiology across several species, as described, for instance, between horses and ponies [144], and cats and dogs [145]. Notwithstanding, the published case reports suggest that gas plasma technology work regardless of the mammal species investigated so far.

## 5. Conclusions

The ROS/RNS-generating technology of gas plasmas is highly auspicious in modulating the inflammatory state of wounds to promote healing. Experimental gas plasma-assisted wound healing models have helped to close significant knowledge gaps on molecular mechanisms and favorable physical plasma conditions. Open questions relate to gas plasma effects on the detrimental contribution of different microbial strains, including drug-resistant bacteria, in rendering wound healing ineffective. Apart from supporting wound healing in humans, a highly promising gas plasma application relates to defective healing in veterinary medicine, especially for pets and animals engaged in sports events.

## Figures and Tables

**Table 1:** Study overview on gas plasma-treated skin and wound healing in mice.

Animal Model	Discharge Type	Key Outcomes	**Ref.**
**Wounded**			
129Sv/Ev	Ar plasma torch system	Elevated FGF-2 production and angiogenesis	[58]
129Sv/Ev	Ar plasma torch system	Faster wound closure; elevated neutrophil and macrophage levels; higher production of IL-6 and MCP-1; collagen type I production increased	[57]
Balb/c	He plasma jet or treated liquid	Direct gas plasma exposure did not improve wound healing; gas plasma-treated liquid did not improve healing	[59]
Balb/c	Ar or He plasma jet	Accelerated wound healing; faster hemostasis	[38]
Balb/c	He plasma jet	Improved vascularization and angiogenesis	[53]
Balb/c	Ar plasma jet or treated water	Reduced feed gas flux increased wound temperature; direct gas plasma treatment or exposure to gas plasma-treated water improved wound healing; elevated numbers of myofibroblast	[60]
Balb/c	Ar plasma jet	Promotion of inflammation, re-epithelization, and wound contraction	[37]
Balb/c	DBD or Ar or He plasma jet	Ar and He plasma jets showed best wound healing promotion; DBD plasma showed good wound healing promotion	[56]
Balb/c	He plasma jet	Improved wound healing	[52]
Balb/c	Ar plasma jet	Wound healing not compared; exposure time-dependent changes of collagen and vimentin	[47]
Balb/c	Ar plasma jet	Improved wound healing for short but not long exposure times; long treatment showed necrosis and inflammatory cell influx	[45]
Balb/c	He/O_2_/N_2_ plasma jet	Accelerated neovascularization and epithelization; decreased microbial burden of natural wound flora	[54]
Balb/c	He plasma jet	Skin grafts on wounds exposed to gas plasma elevated angiogenesis and CD31 and hemoglobin expression; increased VEGFR2, PDGFRβ, and eNOS; less TSP-1 expression	[61]
Balb/c	Ar plasma jet	Short remote but not long direct exposure promoted wound healing and re-epithelization	[46]
Balb/c	Ar plasma jet + medical honey	Lack of beneficial effect of the combination of gas plasma with hydrocolloid dressings and medical honey	[62]
Balb/c	Ar plasma jet + medical honey	No improved healing	[63]
Balb/c	Plasma jet	Infection model; gas plasma jet-to-wound contact reduced infection while remote gas plasma jet treatment was better to stimulate wound healing	[55]
Balb/c	Hot He plasma jet	This gas plasma jet reached up to 90 °C and hence damaged the skin under various feed gas fluxes and treatment times	[64]
BKS.CG	He plasma jet	Improved wound closure in diabetic mice with moderate gas plasma treatment; short or long exposure did not improve healing as much	[65]
C57/BL6	N_2_ plasma jet	Wound healing rates dependent on times points investigated; elevated secondary RNS in wound tissue; improved angiogenesis; earlier epithelization and wound contraction	[50]
C57/BL6	N_2_/air plasma jet	Improved wound contraction and healing	[49]
C57/BL6	N_2_/Ar plasma jet	Repeated but not single exposure increased wound healing; elevated blood flow and RNS deposition into tissue; augmented wound strength and laminin production; decreased MMP3	[51]
C57/BL6	Air/He DBD	Treatment of scars two weeks after wounding led to reduction of scar tissue, thickness, and vascularization	[66]
C57/BL6	Plasma jet-treated liquid for hydrogels	Gas plasma-treated liquid enriched in hydrogel improved wound healing in random-pattern skin-flap full-thickness wounds	[67]
C57/BL6	Ar plasma jet	Improved healing in sterile and infected burn wounds; lower TNFα levels; bacterial burden unchanged	[68]
db/db	He or He/O_2_ plasma jet	Faster wound healing in a diabetic model, predominantly in He/0.1% O_2_ compared to He and He/1% O_2_ plasma; elevated bFGF and VEGF in all treatment groups investigated	[69]
ICR	Air DBD-treated water	Improved wound healing; enhanced antimicrobial efficacy; altered wound microbiome	[70]
ICR	DBD-treated water	Improved wound healing; enhanced pro-inflammatory and anti-inflammatory cytokines and growth factors	[71]
SKH-1	Ar plasma jet	Elevated wound tissue oxygenation in deep and superficial layers; enhanced tissue hemoglobin and water index	[39]
SKH-1	Ar or Ar/air plasma jet	Best wound closure in and IL-6 mRNA in Ar/Air over Ar plasma	[43]
SKH-1	Ar/air plasma jet	Dual-frequency but not single-frequency and 2 but not 1, 3, or 4 gas plasma treatment cycles improved healing; scab hampered gas plasma effects	[44]
SKH-1	Ar plasma jet	Improved angiogenesis and wound closure	[36]
SKH-1	Ar plasma jet	Lack of adverse events or cancerogenesis one year after repeated gas plasma exposure; improved wound healing	[42]
SKH-1	Ar plasma jet	Improved angiogenesis and wound healing; faster re-epithelization; elevated keratin production and collagen fibers; augmented p53 activation, Nrf2 response, macrophage infiltration, inflammation, and granulation	[40]
SKH-1	Ar plasma jet	Focal adhesion signaling complexes and junctional protein expression along with EMT and FMT accompanied improved wound healing	[41]
(not stated)	Ar/O_2_ plasma-treated oil emulsion	Improved wound closure	[72]
(not stated)	Ar/air or Ar/O_2_ plasma jet-treated oil	Accelerated wound healing with oil application previously treated with either air or O_2_ plasma regimens	[73]
(not stated)	Plasma jet	Improved wound healing and formation of keratin and granular layers along with collagen and αSMA deposition	[48]
**Non-Wounded**			
129Sv/Ev	Ar plasma torch	Keratinocyte proliferation, migration, and apoptosis did not change; β-defensins were found upregulated	[74]
Balb/c	He/O_2_ plasma jet	Ameliorated morphological manifestation and reduced epidermal proliferation in imiquimod-induced psoriatic lesions	[75]
Balb/c	Air DBD	Increased NO deposition; lack of inflammation or changes in skin collagen	[76]
Balb/c	Ar plasma jet	Local treatment through healthy skin in diabetic mice; presumable systemic effects on MDA, AOPP, oxLDL, and cytokines observed	[77]
Balb/c	Ar/N_2_/O_2_/humidity DBD	Allergic contact dermatitis models; humidified argon decreased disease severity greater than N_2_ or O_2_ admixture	[78]
C57/BL6	N_2_ plasma jet	Imiquimod-induced psoriasis-like inflammation; reduced epidermal thickness, leukocyte infiltrate, chemokine/cytokine expression (IL-6, IL-17, IL-22, CXCL1, CCL20)	[79]
CBA	Ar plasma jet	Lack of allergic response in intact skin	[80]
HRM-2	Ar plasma jet	E-cadherin decreased; EGF absorption through skin increased	[81]
HRM-2	Ar DBD	Enhanced TGF-β, VEGF, GM-CSF, and EGF levels, and epidermal thickness	[82]
HRS	Ar plasma jet	No dermal degeneration in intact skin; lack of follicular atrophy; elevated catalase and Nrf2 expression	[83]
KM	Air DBD	Increased lidocaine drug absorption in gas plasma-treated skin; increased permeability was reversible 30 min post-treatment	[84]
NC/Nga	N_2_ DBD	Atopic dermatitis-like allergic skin inflammation model; less recruited mast cell and eosinophils; decreased epidermal thickness; less T_H_2 differentiation	[85]
SKH-1	Ar plasma jet	Increased uptake of model drug and tissue oxygenation and microcirculation; changes in lipid composition; changes in junctional protein expression	[86]
SKH-1	Plasma jet-treated water	Decrease of symptoms in a model of atopic dermatitis; decreased catalase and increased SOD activity	[87]
(not stated)	He/Ar/N_2_/air plasma jet	Psoriasis model; effectiveness in treating psoriasis shown	[88]

**Table 2:** Study overview on gas plasma-treated skin and wound healing in medium-sized rodents (rats, rabbits).

Animal Model	Discharge Type	Key Outcomes	**Ref.**
Rats	He plasma jet	In non-diabetic and diabetic rats, improved wound healing, acute inflammation, and neovascularization	[94]
Sprague Dawley rats	Ar plasma jet	Increased re-epithelization and wound closure; more acute inflammation and less fibrosis	[89]
Sprague Dawley rats	Ar plasma jet	Faster wound closure in rats as well as Type I and Type II diabetic rats; accelerated re-epithelization and fewer neutrophils; elevated SOD, catalase, and GPx in treated tissues	[90]
Sprague Dawley rats	Ar plasma jet	Decelerated burn wound injury progression regarding necrosis and neutrophil influx	[91]
Sprague Dawley rats	He/Ar plasma jet	Improved granulation tissue formation and wound healing; He/Ar better than He plasma; short treatment time better than intermediate and long treatment times	[92]
Sprague Dawley rats	He plasma jet	Decreased collagen deposition, scar formation, and TGF-β/pSmad2/pSmad3/αSMA positive cells; improved healing	[97]
Sprague Dawley rats	He plasma jet	Improved wound contraction, healing, and epithelization; unchanged collagen; increased TNFα and IL-1β but not IL-10	[96]
Wistar rats	He plasma jet	Improved wound closure, angiogenesis, re-epithelization, inflammation; stronger force resistance; elevated elastic stiffness	[95]
Wistar rats	He plasma jet	Elevated expression of interleukin-6, nitric oxide synthase 2, prostaglandin-endoperoxide synthase 2 mRNA; decreased expression of nuclear factor ‘kappa-light-chain-enhancer’ of activated B-cells and superoxide dismutase 1 mRNA; improved wound closure speed	[93]
Wistar rats	Ar DBD	Elevated angiogenesis, free thiols, leukocyte influx, and MPO release	[98]
Wistar rats	Air DBD	Augmented healing of non-infected and infected wounds; elevated angiogenesis and granulation; shortened inflammation	[99]
Wistar rats	Air DBD	Attenuated long-term inflammation and improved wound healing and collagen I/collagen III ratio	[100]
Wistar rats	Air DBD	Improved healing in diabetic rats; increased epidermal thickness, re-epithelization, collagen deposition, angiogenesis, proliferation, TGF-β, and fibroblasts counts; decreased T cells	[101]
Wistar rats	Air DBD-functionalized PEG	Gas plasma-increased adhesion of polypropylene to absorb betaine hydrochloride, which improved wound healing in diabetic rats	[102]
New Zealand white rabbits	He DBD	Removal of MRSA in infected wounds; modulation of cytokine secretion, inflammation; accelerated re-epithelization and healing	[103]
New Zealand white rabbits	Air DBD	Lower leukocyte infiltration and decreased viability when investigated early after gas plasma treatment	[104]
New Zealand white rabbits	Plasma jet	Gas plasma treatment of the skin in diabetic animals was proposed to alter the tender structure in joints	[105]

**Table 3:** Study overview on gas plasma-treated skin and wound healing in other mammals and pet patients.

Animal Model	Discharge Type	Key Outcomes	**Ref.**
**Large Animal Models**			
Bergamasca sheep	He plasma torch	Improved wound healing; lower microbial wound burden; elevated proliferation and VEGF during early wound healing phases	[110]
Bergamasca sheep	He plasma torch	Improved wound healing efficacy when combined with the application of mesenchymal stem cells	[111]
Pigs	Ar plasma jet	Both superficial dermal wounds and deep wounds healed well following gas plasma treatment with no side effects; compared to untreated wounds, there were fewer inflammatory (immune) cells in fully healed gas plasma-treated wounds	[9]
Pigs	Air DBD	Intact skin was damaged with extended exposure times; moderate energy deposition/exposure time did not harm tissue; blood coagulation observed	[112]
**Animal Patients**			
Dog (case report)	Ar plasma jet	Cutaneous infection with *Alternaria* spp. was alleviated; cessation of immunosuppressive drugs; complete clinical remission	[113]
Dogs	Ar plasma jet	Study in 40 dogs with canine bite wounds revealed no superior antimicrobial efficacy of gas plasma; gas plasma exposure was void of side effects	[114]
Dogs	Ar plasma jet	Study in 85 dogs with canine bite wounds revealed no superior antimicrobial efficacy of gas plasma; gas plasma exposure was void of side effects	[115]
Dogs and cats	Ar plasma jet	8 case studies using pets suffering from chronic wounds since 1, 2, 6, 24, 36, 48, 60, and 80 months; complete healing achieved in 7 out of 8 patients	[116,117]
Dogs, cats, Guinea pigs	Ar plasma jet	12 case studies using pets suffering from insufficient wound healing; all 12 patients experienced complete remission with repeated gas plasma treatment	[116,118]

## Data Availability

Not applicable.
